# T1-weighted gradient-echo imaging, with and without inversion recovery, in the identification of anatomical structures on the lateral surface of the brain*

**DOI:** 10.1590/0100-3984.2015.0033

**Published:** 2016

**Authors:** Sergio Murilo Georgeto, Carlos Alexandre Martins Zicarelli, Munir Antônio Gariba, Luiz Roberto Aguiar

**Affiliations:** 1 Neurosurgeon at the Irmandade da Santa Casa de Londrina and in the Department of Neurosurgery at the Universidade Estadual de Londrina (UEL), Londrina, PR, Brazil; 2 Professor in the Graduate Program in Health Technology at the Pontifícia Universidade Católica do Paraná (PUCPR), Curitiba, PR, Brazil

**Keywords:** Magnetic resonance imaging, Brain/anatomy & histology, Reproducibility of results

## Abstract

**Objective:**

To compare brain structures using volumetric magnetic resonance imaging with isotropic resolution, in T1-weighted gradient-echo (GRE) acquisition, with and without inversion recovery (IR).

**Materials and methods:**

From 30 individuals, we evaluated 120 blocks of images of the left and right cerebral hemispheres being acquired by T1 GRE and by T1 IR GRE. On the basis of the Naidich et al. method for localization of anatomical landmarks, 27 anatomical structures were divided into two categories: identifiable and inconclusive. Those two categories were used in the analyses of repeatability (intraobserver agreement) and reproducibility (interobserver agreement). McNemar's test was used in order to compare the T1 GRE and T1 IR GRE techniques.

**Results:**

There was good agreement in the intraobserver and interobserver analyses (mean kappa > 0.60). McNemar's test showed that the frequency of identifiable anatomical landmarks was slightly higher when the T1 IR GRE technique was employed than when the T1 GRE technique was employed. The difference between the two techniques was statistically significant.

**Conclusion:**

In the identification of anatomical landmarks, the T1 IR GRE technique appears to perform slightly better than does the T1 GRE technique.

## Abstract (pt)

**Objetivo:**

Comparar os resultados de identificação de estruturas cerebrais utilizando imagens volumétricas isotrópicas por ressonância magnética, nas aquisições T1 GRE e T1 IR GRE.

**Materiais e métodos:**

Foram avaliados 120 blocos de imagens, de 30 indivíduos, com imagens extraídas dos hemisférios cerebrais esquerdo e direito, pelos dois métodos de aquisição: T1 GRE e T1 IR GRE. Com base no método de Naidich et al. para localização dos referenciais anatômicos, 27 estruturas anatômicas foram classificadas em duas categorias - identificável versus deixam dúvidas quanto à identificação somadas às não identificáveis - para análises de repetitividade (intraobservador) e reprodutibilidade (interobservadores). Foi utilizado o teste de McNemar para a avaliação do desempenho entre os dois métodos.

**Resultados:**

Após confirmação de ter havido boa concordância na análise intraobservador e interobservadores (kappa médio > 0,60), a avaliação das imagens de cada referencial anatômico, testada entre T1 GRE e T1 IR GRE pelo teste de McNemar, indicou maior frequência de referenciais identificáveis pelo método T1 IR GRE do que pelo método T1 GRE.

**Conclusão:**

O método de imagem T1 IR GRE apresentou desempenho levemente superior, porém estatisticamente significante, em relação ao método T1 GRE, na identificação dos referenciais anatômicos cerebrais.

## INTRODUCTION

Currently, the topographic anatomy of the brain can be described as much by computed tomography (CT) as by magnetic resonance imaging (MRI). The new CT scanners, with multiple detectors, have good spatial resolution and make it possible to draw correlations with craniometric points. The disadvantages of those CT scanners are the higher dose of radiation emitted and the lower resolution of images of the cortical mantle^(1)^. Studies comparing CT and MRI in terms of their ability to discern the anatomical structures of the lateral surface of the brain have shown that T1-weighted, spinecho MRI sequences are superior to CT scans for the identification of predetermined structures^(2)^.

Because there are no suitable bone landmarks to facilitate the MRI localization of the elements of the lateral surface of the brain, a system of identifying sulci and gyri was developed in order to study normal anatomical relationships. The use of a sagittal section of the lateral fissure at its deepest extent, together with the application of the method for identification of sulci and gyri on the lateral surface, has been found to be particularly successful in the characterization of preselected anatomical elements in T1- and T2-weighted spin-echo MRI sequences^(3)^.

Recent advances in techniques of acquisition and post-processing of images have allowed the acquisition of T1-weighted gradient-echo and T1-weighted gradient-echo with inversion recovery (T1 GRE and T1 IR GRE, respectively) pulse sequences to be employed in routine exams, the capture times no longer representing a financial hindrance to their use. With the improvement in coils and image post-processing methods, it became possible to obtain images from three-dimensional matrices of isotropic voxels, which improved the quality of reconstructions in any orthogonal plane. These images are referred to as volumetric sequences with isotropic resolution^(4)^.

The images obtained by T1 GRE have routinely been used in order to demonstrate changes in the cortical topography in most MRI studies. However, the T1 IR GRE images provide better contrast between gray and white matter, allowing greater accuracy in the identification of anatomical landmarks^(5)^.

The present study aimed to evaluate the identification of anatomical landmarks, using MRI scans of the lateral surface of the brain, with pulse sequences that can currently be used for topographic localization. The T1 GRE and T1 IR GRE weightings were selected because the former is routinely used in most MRI scans and the latter is not usually indicated for the evaluation of structures of the cortical mantle. If the T1 IR GRE technique could be shown to be superior to that of T1 GRE, it would be a useful finding, because anatomical topography studies of the sulci and gyri that comprise the cerebral cortex are of interest not only for professionals involved in the diagnosis and treatment of diseases affecting these areas, in terms of the practical aspect of their daily routine^(6)^, but also for neuroscientists who draw anatomical and functional correlations between cortical patterns and the development of diseases^(7)^.

The objective of this study was to analyze the performance of pulse sequences obtained with the T1 GRE and T1 IR GRE techniques. To that end, it was necessary to assess, initially, the reliability of the techniques for the method chosen, through analysis of intraobserver and interobserver agreement, and subsequently to compare the performance of the two techniques in order to show which might be more capable of identifying the anatomical landmarks of the lateral surface of the brain.

## MATERIALS AND METHODS

The study was approved by the Research Ethics Committee of the Pontifícia Universidade Católica do Paraná. All participating subjects gave written informed consent. In an initial interview, potentially eligible participants completed a questionnaire that included a hand dominance inventory (the Edinburgh handedness inventory).

Subjects who showed any neurological changes were excluded, as were those with any other clinical conditions that would preclude the examination. After the initial interview, eligible individuals were referred for MRI at a scheduled day and time. The sample consisted of 30 young adults, with a mean age of 25.3 years; 16 (53.3%) were female, and 14 (46.7%) were male.

The MRI scans were obtained in a 1.5 T scanner (Magnetom Symphony; Siemens, Erlangen, Germany), with 12-channel coil. The volumetric sequences with isotropic resolution were obtained with T1 GRE and T1 IR GRE sequences. For the T1 GRE sequence, we used sagittal gradient-echo volumetric acquisition, with a 256 × 256 matrix, isotropic voxel (1 × 1 × 1 mm), repetition time/echo time (TR/TE) of 1910/3.09 ms, field of view (FOV) of 256 mm, slice thickness of 1 mm, no intervals between slices, and a flip angle of 15º. For the T1 IR GRE sequence, we used coronal volumetric acquisition, with a 256 × 256 matrix, isotropic voxel (1 × 1 × 1 mm), TR/TE of 4000/373 ms, FOV of 260 mm, slice thickness of 1 mm, no intervals between slices, and an inversion time of 350 ms. The Magnetom Symphony scanner is equipped with a Quantum gradient (30 mT/m), with a slew rate of 150 mT/ms. A flip angle of 15º was chosen on the basis of data in the literature^(8)^. The remaining parameters have been used routinely at the radiology clinic where the images were obtained and were in accordance with the manufacturer's recommendations.

**Figure 1:**
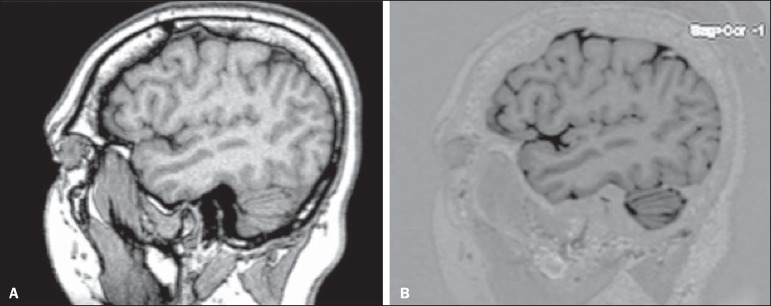
Sagittal images of the lateral fissure at its deepest extent, obtained with the T1 GRE (**A**) and T1 IR GRE (**B**) techniques.

An experienced radiologist analyzed the scans, with the goal of excluding images with motion artifacts or that were inappropriate for evaluation, as well as identifying pathological findings. The files for all 30 T1 GRE- and T1 IR GRE-weighted MRIs of the brain were transferred to OsiriX M.D. software, version 5.7.1, 64-bit (Pixmeo SARL, Bernex, Switzerland). Thus, 60 image blocks were formed, and the right and left hemispheres were analyzed separately, resulting in a total of 120 blocks. The images obtained in each of the sequences can be seen in Figure 1.

The anatomy of the lateral surface of the brain was assessed qualitatively as to the identification of the major anatomical structures, and the method described by Naidich et al.^(3)^, which consists in the description of 15 steps (or signs) for the identification of 27 anatomical structures that compose the lateral surface of the brain on MRI slices obtained in the sagittal plane, was used as a reference.

The T1 GRE and T1 IR GRE acquisitions were analyzed in sagittal slices, with two-dimensional reconstructions, thus minimizing the superimposition of sulci and gyri that occurs in the coronal and axial planes. The analysis began with the identification of the lateral fissure at its deepest extent, being then performed the sequence of procedures that comprise the 15 steps in the method, with the purpose of identifying the 27 anatomical structures on the lateral surface of the brain, as described in the most recent study conducted by Naidich et al.^(3)^. For the identification of structures, three categories were considered (Table 1): easily identifiable; inconclusive; and unidentifiable.

**Table 1:** Anatomical landmarks to be identified on the lateral surface of the brain.

	Anatomical structures	Easily identifiable	Inconclusive	Unidentifiable
Step 1	1. The lateral convexity (sagittal view) in the segment where the deepest extent of the lateral fissure can be seen			
Step 2	2. Lateral fissure			
	2.1. Posterior horizontal ramus			
	2.2. Anterior horizontal ramus			
	2.3. Anterior ascending ramus			
	2.4. Posterior ascending ramus			
	2.5. Posterior descending ramus			
	2.6. Anterior subcentral sulcus			
	2.7. Posterior subcentral sulcus			
	2.8. Transverse temporal sulcus			
	2.9. Anterior sylvian point			
Step 3	3. Inferior frontal gyrus			
	3.1. Orbital part			
	3.2. Triangular part			
	3.3. Opercular part			
Step 4	4. Inferior frontal gyrus			
Step 5	5. Connection between the middle frontal and precentral gyrus			
Step 6	6. Precentral sulcus			
Step 7	7. Precentral gyrus			
Step 8	8. Central sulcus			
Step 9	9. Postcentral gyrus			
Step 10	10. Postcentral sulcus			
Step 11	11. Posterior ascending ramus of the lateral fissure/supramarginal gyrus			
Step 12	12. Superior temporal sulcus			
Step 13	13. Angular gyrus			
Step 14	14. Intraparietal sulcus			
Step 15	15. Superior parietal lobe			

*Source*: Modified from Naidich et al.^(3)^.

Observer 1 analyzed the repeatability of the 120 image blocks in triplicate, with a minimum interval between observations of 10 days, and the observation sequences of the groups of 120 images were randomly recoded at each time point. That resulted in 360 observations (30 individuals × 2 cerebral hemispheres × 2 imaging techniques × 3 evaluations) for the intraobserver analysis.

Two other evaluators (observers 2 and 3) analyzed the anatomical structures. The observers were practicing neurosurgeons and were invited to participate because of their familiarity with the anatomy and corresponding images of the region in question. Those procedures resulted in 240 additional observations (30 individuals × 2 cerebral hemispheres × 2 imaging techniques × 2 observers). However, in the reproducibility analysis, we also used the first assessment of the researcher, resulting in another 120 comments. Therefore, a total of 360 observations were used in the interobserver analysis.

All of the observers received an instruction manual with relevant information about the variables to be assessed, an explanation of its use, and tables in which to note their findings. The image sequences were all displayed in an standardized manner on an Apple MacBook Pro notebook with a 15" screen with retina display, 2880 horizontal pixels, 1800 vertical pixels, and 220 ppi resolution. The sequence of 120 image blocks was randomly reordered in three different modes, and each observer had access only to their own data sequence.

The performance of the T1 GRE and T1 IR GRE imaging techniques was assessed in two stages: we initially ascertained whether the two techniques led the observers to similar results, as determined by analysis of agreement based on the kappa index, and subsequently established the agreement of results between the observers. We then attempted to determine whether either technique was able to identify the anatomical structures more easily than was the other. To that end, we used McNemar's nonparametric test. For the image processing in the second stage, six different scenarios were taken into account.

## RESULTS

In the analysis of intraobserver and interobserver agreement, weighted kappa statistics were estimated for each of the three categories: easily identifiable; inconclusive; and unidentifiable. Of the 216 potential kappa statistics-for 27 anatomical structures, in duplicate (right and left sides); for T1 GRE and T1 IR GRE sequences; and for the intraobserver and interobserver analyses-it was possible to estimate 115: 58 from the intraobserver analysis and 57 from the interobserver analysis.

In general, all kappa statistics were significant at the 1% level, indicating that the agreement among virtually all evaluations was positive and different from zero. Only one kappa statistic-for the interobserver analysis of the performance of the T1 GRE technique in identifying the anterior subcentral sulcus on the right side-was less than statistically significant (0.04). The mean of the kappa statistics calculated was 0.62 ± 0.02, which is considered indicative of good agreement^(9)^.

According to the criteria established by Byrt et al.^(9)^, only 24.3% of our kappa statistics were classified as poor (< 0.2) or weak (< 0.4). There were no statistical differences between the intraobserver and interobserver analyses: the mean kappa was 0.60 ± 0.03 for the intraobserver analysis and 0.63 ± 0.04 for the interobserver analysis.

After good intraobserver and interobserver agreement had been confirmed, McNemar's test was carried out in order to determine whether either technique was superior to the other in its ability to identify the anatomical structures under study. In conducting McNemar's test, we assumed that each evaluation of an image of an anatomical structure was independent of the other. Our hypothesis that the evaluations were independent can be considered strong because of the characteristics of the study data, such as individual evaluators, individual images, and different time points, which could indicate dependency among the cases. However, if the results remain stable in the various simulations, one could conclude, on the basis of the evidence, in favor of the findings obtained.

Theoretically, there would have been 16,200 observations: 8,100 for the T1 GRE technique and 8,100 for the T1 IR GRE technique. That is because the observations collected-3 (from observer 1) + 1 (from observer 2) + 1 (from observer 3) = 5 × 120 image blocks = 600-were multiplied by the number of anatomical structures analyzed (27 × 600 = 16,200) and divided by the number of techniques evaluated (16,200 / 2 = 8,100). However, two anatomical landmarks-the lateral convexity and the sylvian fissure-were excluded from the results because they presented 100% agreement in the intraobserver and interobserver analyses. That was done so as not to overestimate the frequencies of identifiable images, given that those two structures are easily identifiable with any technique. Consequently, only 15,000 observations (7,500 each for the T1 GRE and T1 IR GRE techniques) were evaluated.

Those observations were submitted to McNemar's test in six scenarios: 1) all observations (*n* = 15,000); 2) only the observations considered in the intraobserver analysis (*n* = 9,000); 3) only the observations considered in the interobserver analysis (*n* = 9,000); 4) only the first observations made by observer 1 (*n* = 3,000); 5) only the observations made by observer 2 (*n* = 3,000); and 6) only the observations made by observer 3 (*n* = 3,000).

For the application of McNemar's test, we combined two of the three structure identification categories and therefore considered only two: easily identifiable (identified); and unidentifiable + inconclusive (unidentified). In practical terms, those two categories are the ones that matter: conclusively identifying the anatomical structure or not. For McNemar's test to be applied, there must be only two categories. In essence, we tested the difference in the frequency of anatomical structures categorized as identifiable by comparing a group resulting from the sum of the frequency of those categorized as unidentifiable, between the T1 GRE and T1 IR GRE techniques, in six different scenarios. It should be borne in mind that the unidentified group is the sum of the frequencies of structures categorized as (identification) inconclusive and of those categorized as unidentifiable. Those frequencies are summarized in Table 2.

**Table 2:** Absolute and relative frequencies of images in which the target structures were identified (easily identifiable) or unidentified (unidentifiable + inconclusive) with the T1 GRE and T1 IR GRE techniques, in six different scenarios.

			T1GRE		
Scenario	T1 IR GRE		Unidentifiable + inconclusive	Identifiable	Total
1) All observations (*n* = 15,000)	Unidentifiable + inconclusive	*n*	582	90	673
		%	7.8%	1.2%	9.0%
	Identifiable	*n*	256	6,571	6,827
		%	3.4%	87.6%	91.0%
	Total	*n*	838	6,662	7,500
		%	11.2%	88.8%	100.0%
2) Intraobserver analysis (n = 9,000)	Unidentifiable + inconclusive	*n*	374	53	427
		%	8.3%	1.2%	9.5%
	Identifiable	*n*	176	3,897	4,073
		%	3.9%	86.6%	90.5%
	Total	*n*	550	3,950	4,500
		%	12.2%	87.8%	100.0%
3) Interobserver analysis (*n* = 9,000)	Unidentifiable + inconclusive	*n*	341	51	392
		%	7.6%	1.1%	8.7%
	Identifiable	n	145	3,963	4,108
		%	3.2%	88.1%	91.3%
	Total	n	486	4,014	4,500
		%	10.8%	89.2%	100.0%
4) First evaluation of observer 1 (*n* = 3,000)	Unidentifiable + inconclusive	*n*	133	13	146
		%	8.9%	0.9%	9.7%
	Identifiable	*n*	65	1,289	1,354
		%	4.3%	85.9%	90.3%
	Total	*n*	198	1,302	1,500
		%	13.2%	86.8%	100.0%
5) Evaluation of observer 2 (*n* = 3,000)	Unidentifiable + inconclusive	*n*	103	23	126
		%	6.9%	1.5%	8.4%
	Identifiable	*n*	48	1,326	1,374
		%	3.2%	88.4%	91.6%
	Total	*n*	151	1,349	1,500
		%	10.1%	89.9%	100.0%
6) Evaluation of observer 3 (*n* = 3,000)	Unidentifiable + inconclusive	*n*	105	15	120
		%	7.0%	1.0%	8.0%
	Identifiable	*n*	32	1,348	1,380
		%	2.1%	89.9%	92.0%
	Total	*n*	137	1,363	1,500
		%	9.1%	90.9%	100.0%

*n*, absolute frequency; %, relative frequency. Scenario 1 (McNemar: χ^2^ = 77.51; *p*-value = 0.000); Scenario 2 (McNemar: χ^2^ = 64.97; *p*-value = 0.000); Scenario 3 (McNemar: χ^2^ = 44.12; *p*-value = 0.000); Scenario 4 (McNemar: χ^2^ = 33.35; *p*-value = 0.000); Scenario 5 (McNemar: χ^2^ = 8.11; *p*-value = 0.003); Scenario 6 (McNemar: χ^2^ = 5.48; *p*-value = 0.020).

Of the identifiable images in all observations, 91% were identifiable with the T1 IR GRE technique and 88.8% were identifiable with the T1 GRE technique (McNemar's χ^2^ = 77.51; *p* = 0.000), indicating that the T1 IR GRE technique features significantly (albeit only slightly) better performance than does the T1 GRE technique.

The evidence of superior performance of the T1 IR GRE technique remained robust across all of the scenarios considered. In all six scenarios, McNemar's test showed at least 5% statistical significance. In the first evaluation of the researcher, for example, the difference between the T1 GRE and T1 IR GRE techniques in terms of the frequency of identifiable structures was 3.5%, which is highly significant. If all of the independent evaluations are considered, as typically occurs in the evaluation of MRI scans, it can be concluded that the T1 IR GRE technique performs slightly better than does the T1 GRE technique.

## DISCUSSION

There is no established MRI technique for the assessment of the elements that compose the cortical mantle. Various types of pulse sequences have been described in the literature: T1 GRE^(10)^; T2 GRE^(11)^; spoiled gradient-recalled acquisition in steady state^(12)^; spoiled GRE^(13)^; and T1 IR GRE^(14)^. GRE-weighted images, obtained by matrices of isotropic voxels enable high resolution in any of the orthogonal planes selected for reconstruction^(5)^.

Acquisitions in T1 IR GRE provide better contrast between gray and white matter in the convolutions of the gyri, because the water contained within the cortical region, which is concentrated mainly in the cytoplasm of neurons and glial cells, presents greater signal strength when inversion recovery is used in order to form the signal that will produce the image^(15)^. That enables multiple relevant clinical applications, such as better visualization of cryptogenic neocortical lesions and areas of atrophy in the hippocampus, both of which are associated with temporal lobe epilepsy^(16)^. The T1 IR GRE technique has also been shown to be useful for the detection of cortical inflammatory lesions in patients with multiple sclerosis^(14)^.

The contrast that the T1 IR GRE technique creates between the white and gray matter in the cerebral cortex makes it possible to discriminate between the structure and the cerebrospinal fluid in an efficacious manner, which would facilitate the process of segmentation for volumetric studies^(17)^. In a meta-analysis of factors that influence the volumetric analysis of the amygdala by MRI, it was demonstrated that the main factor responsible for volume differences was a lack of precision in the definition of the anatomical region^(18)^. Therefore, a technique that precisely delineates the borders of the chosen landmark in volumetric anatomical studies can facilitate its correct description, making the results more consistent across studies.

Given those expectations, the present study aimed to assess whether, in practical terms, the T1 IR GRE technique features better performance in identifying the 27 anatomical structures that compose the lateral surface of the brain, based on the method described by Naidich et al.^(3)^, than does the T1 GRE technique. On the basis of the materials and methods adopted, primarily the use of five evaluations of 30 MRI scans of the brain with each of the techniques and of both cerebral hemispheres, we can conclude that the performance of the T1 IR GRE technique was statistically better than was that of the T1 GRE technique, despite the fact that the differences were slight.

Nevertheless, this study has limitations that must be taken into consideration. The choice of sequences was based on their routine use for the identification of anatomical landmarks of the cortical mantle, their quality being evidenced by daily use. The comparison of the T1 GRE and T1 IR GRE protocols in order to characterize the signal-to-noise ratio and contrast-to-noise ratio (quantitative analysis) and the inclusion of visual quality criteria (qualitative analysis) would have made the results more robust. Despite the fact that the difference between the techniques was statistically significant, the performance of the T1 IR GRE technique was only slightly superior to that of the T1 GRE technique. Studies using different scanners and including a larger sample are needed in order to corroborate our findings.

## CONCLUSION

We have confirmed that the T1 GRE and T1 IR GRE techniques have good reliability, as evidenced by the weighted kappa statistics for intraobserver and interobserver agreement, in the evaluation of 27 anatomical structures of the brain. McNemar's test showed that the T1 IR GRE technique allows those anatomical structures to be identified more easily than does the T1 GRE technique. For the statistical tests, it was necessary to assume that the evaluation of the images of each of the 27 anatomical references were independent of each other. That hypothesis is not present in the context of the study, because, in essence, it was the same observer, the same image, and the same individual. However, the results remained stable in six simulated scenarios, providing evidence to support the findings obtained. Despite the limitations of the study, the statistical evidence of superior performance of the T1 IR GRE technique over the T1 GRE technique should also be evaluated in practical terms (i.e., cost versus benefit), given that, in one of the simulated scenarios, the T1 IR GRE technique performed only 1.1% better than did the T1 GRE technique. In large samples, as in the case of the use of all of the images for the application of McNemar's test, statistical significance can stand out even without evidence of significant practical differences.
